# Chronic Treatment-Resistant Annular Rash: A Case of Neutrophilic Figurate Erythema

**DOI:** 10.7759/cureus.66920

**Published:** 2024-08-15

**Authors:** Cathleen F Meyer, Rebecca L Johnson, Charles A Sola

**Affiliations:** 1 Aviation Medicine, Naval Medical Center Portsmouth, Portsmouth, USA; 2 Pathology/Dermatopathology, Naval Medical Center Portsmouth, Portsmouth, USA; 3 General Dermatology, Naval Medical Center Portsmouth, Portsmouth, USA

**Keywords:** skin disease/dermatology, dermatopathology, military physician, active-duty military personnel, general dermatology, neutrophilic dermatosis, contact dermatitis, annular erythema

## Abstract

Neutrophilic figurate erythema (NFE) is a rarely reported figurate erythema that clinically presents similarly to erythema annulare centrifugum (EAC) with neutrophil-predominant perivascular and interstitial infiltrate in the dermis on histopathology. We present the case of a 32-year-old active-duty military male who presented with a chronic treatment-resistant skin rash. The rash began on his thighs five years previously and was treated with topical and oral antifungals repeatedly without improvement. The patient was deployed overseas during the rash onset, but the rash persisted upon his return stateside. No triggers were identified. His persistent skin eruption consisted of erythematous polycyclic annular plaques with a "trailing edge" scale. Histologic examination revealed perivascular neutrophils and perivascular and interstitial eosinophils without signs of vasculitis or infection. With only 15 reported cases, it can be difficult to recognize leading to long delays in diagnosis and treatment. Despite having a clinical course similar to EAC, NFE may require anti-neutrophil therapy to resolve completely.

## Introduction

Neutrophilic figurate erythema (NFE) is a rare neutrophil-predominant figurate erythema [1]. NFE is more commonly described in pediatric populations but can also be seen in adults [2]. NFE can present similarly to erythema annulare centrifugum (EAC) on physical exam and is differentiated from EAC on histopathology. Treatment for NFE may differ from EAC and other lymphocyte-predominant figurate erythemas by requiring anti-neutrophil therapy, such as dapsone or colchicine [3]. We present the case of a 32-year-old previously healthy male in the United States Armed Forces who presented to the dermatology clinic for a chronic asymptomatic annular lesion on his right superior inner thigh, waxing and waning in size since 2017.

## Case presentation

History

A 32-year-old previously healthy male in the United States Armed Forces presented to the dermatology clinic for an asymptomatic annular lesion on his right superior inner thigh for five years. He denied any previous significant medical history. He stated that he first noticed the lesion five years ago. He stated that the lesion's appearance remained mostly consistent, but it would occasionally increase or decrease in size but would never fully clear. He denied any pruritus, shortness of breath, fevers, chills, chest pain, night sweats, unexplained weight loss, or infectious symptoms currently and at the onset of the original lesion. He denied personal and family history of similar and other dermatological diseases, malignancies, recent viral or parasitic infections, allergic reactions, arthropod bites, or autoimmune diseases. He denied having a known drug allergy. He denied taking oral medications regularly. Upon his initial presentation to the dermatology clinic, he was given oral terbinafine and topical clotrimazole. He stated the lesion persisted despite both treatments. A physical exam revealed multiple thin erythematous annular plaques with central clearing and trailing white scale on his right inner thigh. He appeared well-developed and nourished. His Fitzpatrick type was 3. No other skin findings were identified on full body skin exam. 

Diagnostic testing

Labs drawn included complete blood count, hepatic function panel, urinalysis, urine protein electrophoresis (UPEP), serum protein electrophoresis (SPEP), human immunodeficiency virus (HIV), antinuclear antibodies (ANA), rapid plasma reagin (RPR), and thyroid-stimulating hormone (TSH), which were within normal limits.

**Figure 1:**
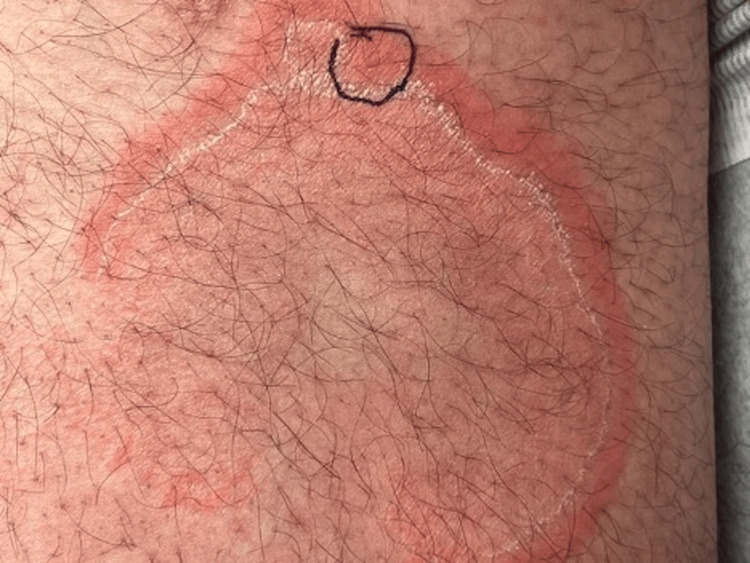
Right inner thigh with large erythematous plaque with trailing white scale and central clearing

**Figure 2:**
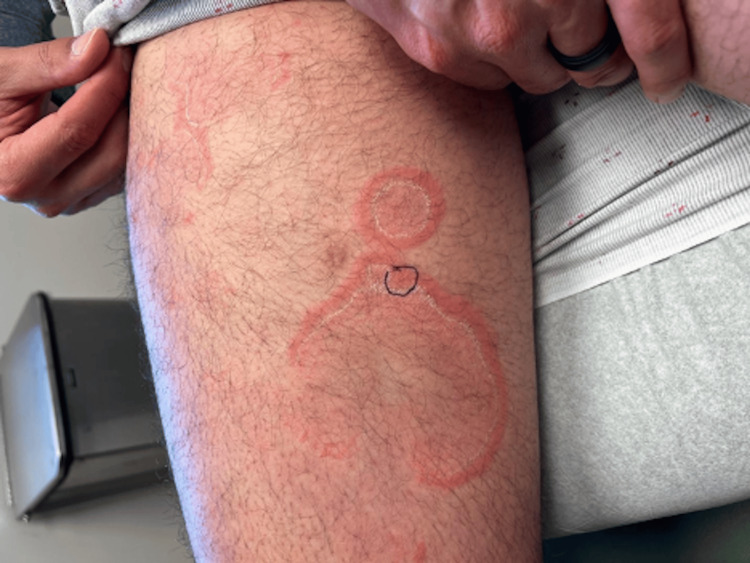
Right inner thigh with a purple circle indicating biopsy site

**Figure 3:**
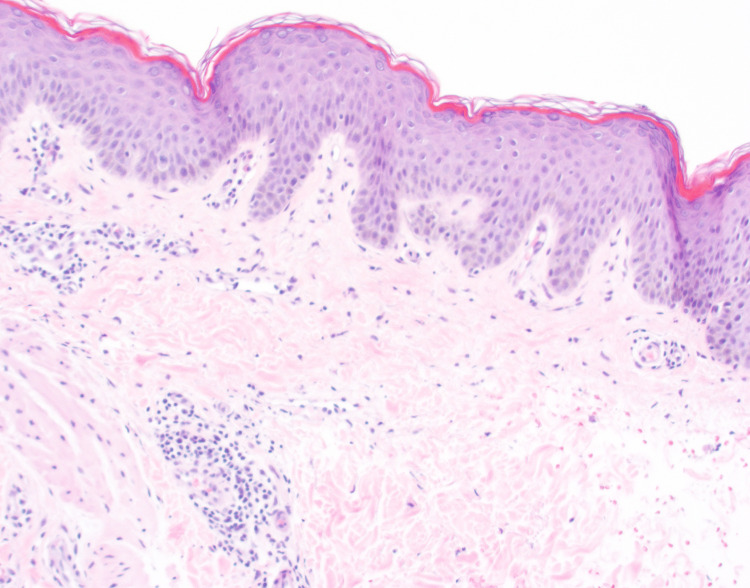
Within the dermis is a perivascular and interstitial inflammatory infiltrate (H&E ×4) H&E: hematoxylin and eosin

Figure 1 and Figure 2 show the location of the biopsy, which was taken at the erythematous rim of the large plaque to capture erythema and trailing edge of scale. The initial biopsy revealed spongiotic dermatitis with occasional eosinophils. Deeper levels and a periodic acid-Schiff with diastase (PASD) stain were evaluated. Sections demonstrated mild spongiosis and a mild perivascular lymphohistiocytic inflammatory infiltrate with rare perivascular and interstitial eosinophils, as shown in Figure 3. The overlying stratum corneum shows variable basketweave pattern and compact orthokeratosis with occasional mounds of parakeratosis and focal neutrophils. The dermis shows superficial red blood cell extravasation. Fresh hemorrhage deep in the specimen is presumed to be procedure-related. No fungal elements are identified on hematoxylin and eosin (H&E) or PASD stains. Changes to the stratum corneum suggest a dermatophyte infection, though no fungal elements are observed on H&E or PASD stains.

**Figure 4:**
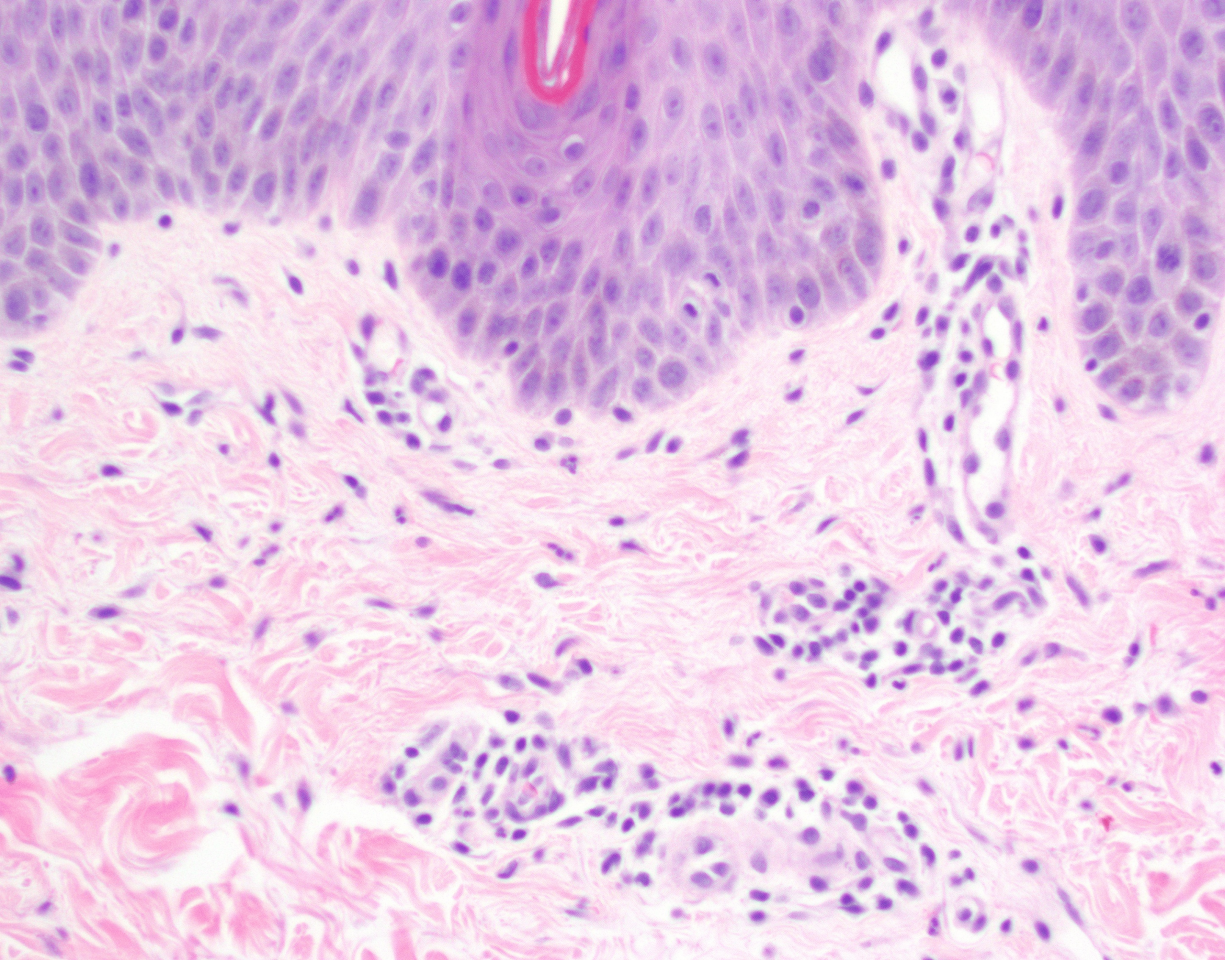
The infiltrate consists of lymphocytes and neutrophils (H&E ×20) H&E: hematoxylin and eosin

**Figure 5:**
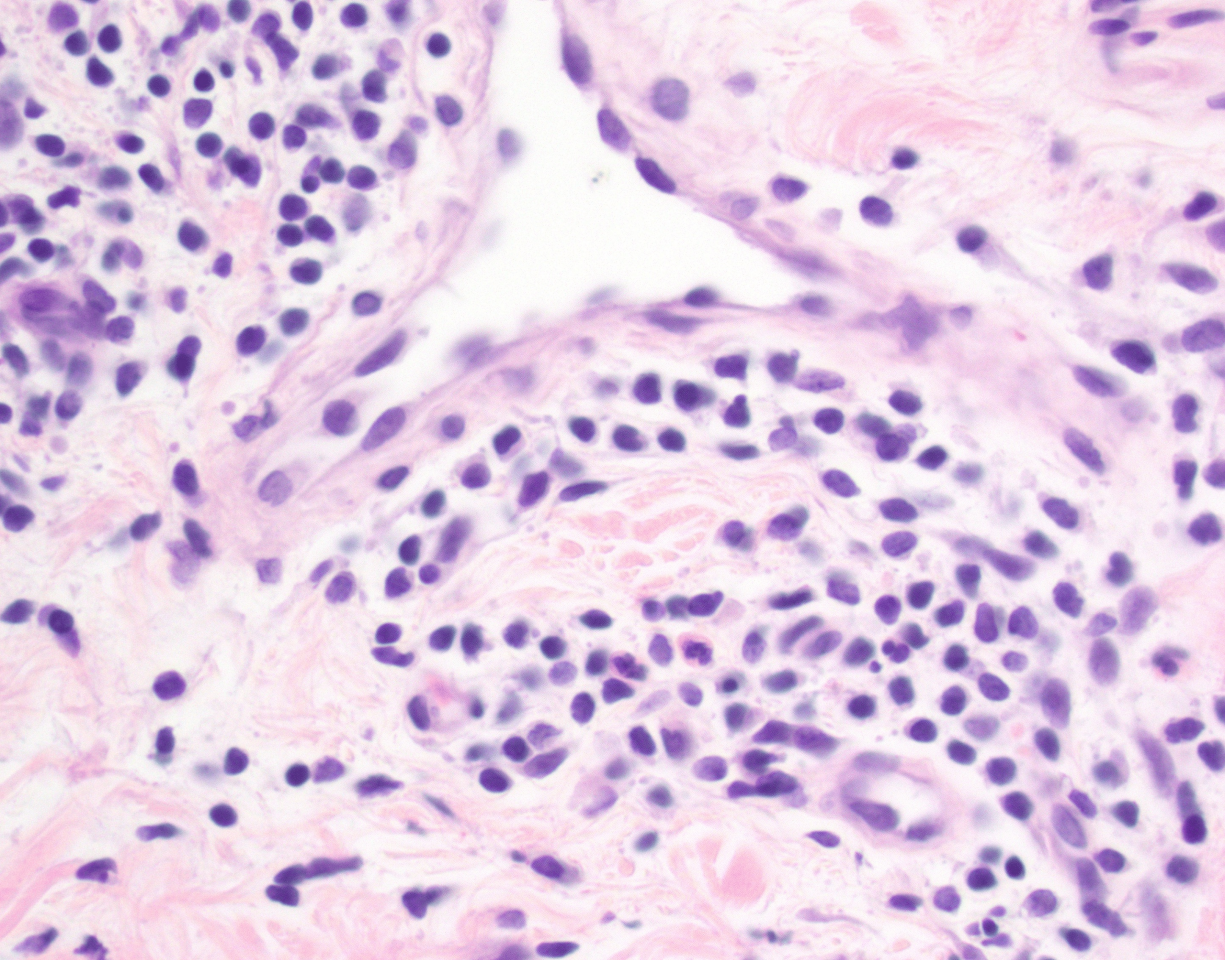
The perivascular infiltrate is mixed with both lymphocytes and neutrophils (H&E ×40) H&E: hematoxylin and eosin

The biopsy specimen was sent for review by dermatopathology. Further examination of the specimens revealed a moderately brisk lymphocytic infiltrate tightly cuffed around superficial blood vessels, best seen on leveled sections in Figure 4. There was focal red blood cell extravasation without vascular damage or leukocytoclasia. Figure 5 demonstrates neutrophils extending into the superficial dermal interstitium and into discrete mounds of parakeratosis in the stratum corneum. The epidermis was mildly spongiotic and alternates between basketweave stratum corneum and mounds of parakeratosis. The submitted stains were reviewed, along with additional stains prepared here. Grocott's methenamine silver stain (GMS) and PASD, received and repeated, were negative for fungal organisms. The Brown and Brenn (B&B) method for gram staining was negative for bacterial organisms. These findings may be seen as a variant of NFE. Biopsy results suggest a diagnosis of NFE.

Differential diagnoses

Differential diagnoses include EAC, which appears clinically similar to NFE. EAC is usually a chronic skin condition, characterized by waxing and waning annular or arcuate erythematous patches, usually found on the trunk or lower extremities. EAC usually occurs secondary to an associated condition, such as infection, drug exposure, or malignancy [4]. However, EAC can also occur idiopathically, without an easily identifiable trigger [5]. EAC can be diagnosed clinically, but tinea infection should be ruled out with a potassium hydroxide scraping. Histopathology findings for EAC typically are nonspecific, showing mild spongiosis with mild superficial perivascular lymphohistiocytic infiltrate and occasional eosinophils [1,5]. This particular distribution of lymphocytes and histiocytes tightly wound around vasculature has been described as a "coat-sleeve" pattern [1]. Two main histopathologic variants of EAC have been identified, which have been described in Fitzpatrick's Dermatology 9e as follows: "The superficial variant consists of spongiosis, parakeratosis, hyperkeratosis or basal vacuolar changes with a perivascular infiltrate, and deep variant consists of perivascular lymphocytic infiltrate concentrated in the upper dermis without any epidermal changes" [1]. In our patient, the biopsy revealed neutrophils extending into the superficial dermal interstitium and into discrete mounds of parakeratosis in the stratum corneum, consistent with previously described cases of NFE [6-8]. Treatment for EAC typically involves removing suspected trigger(s) or treatment of underlying condition(s) and local corticosteroid therapy. However, EAC can be persistent for many months to years with recurrences despite treatment. In a retrospective review of 66 cases of EAC, out of the 27 patients who followed up in the study, five (18.5%) patients had persistent treatment-resistant lesions [9]. Topical steroids in many cases do not induce complete remission of EAC, but they have been shown to improve local inflammation. Other treatments that have been effective include antimicrobials and antifungals, such as azithromycin, erythromycin, fluconazole, and oral metronidazole. It is hypothesized that these drugs may treat subclinical microbial foci that stimulate EAC and the anti-inflammatory properties of some antimicrobial therapies [4]. 

Other possible etiologies for figurate erythemas include eczematous processes, hypersensitivity drug eruption, tinea corporis, annular psoriasis, cutaneous lymphoid hyperplasia, and lymphoma cutis. Various autoimmune disorders can also have erythematous annular, arciform, and polycyclic lesions to include linear IgA bullous dermatosis, Sjörgen's syndrome, and lupus erythematosus [5]. One of the most common causes of figurate erythema includes tinea corporis. This typically presents with an annular lesion having an area of desquamation around the outside of the ring, commonly known as the "leading scale." Conversely, EAC/NFE typically presents with a "trailing scale," or an area of desquamation on the inner rim of the annular plaque. This is a clinically distinguishable feature between superficial tinea infections and EAC/NFE. A retrospective study of NFE conducted by Wu and Hsiao examined the known cases of NFE and categorized them clinically as follows: blistering annular erythema (5/15 patients, 33%), annular erythema with purpuric change (2/15 patients, 13%), purpuric annular erythema with vesicles (4/15 patients, 27%), and multiple annular rash with central ring-shaped scales (4/15 patients, 27%) [6]. Clinical features of NFE were found to be variable, in some cases more consistent with other neutrophilic dermatoses to include Sweet's syndrome, rather than EAC1. 

Another less commonly seen annular plaque is annular psoriasis, which is a milder variant of generalized pustular psoriasis. The disease consists of gyrate, annular lesions with an erythematous, scaly, pustular margin [10]. Typically, annular psoriasis has other common features of psoriasis to include symmetric and generalized distribution and a genetic predisposition. This diagnosis was less likely, as this patient had only one area affected without a family history of dermatological disease.

Treatment and management

The patient was started on triamcinolone 0.1% topical ointment to apply to the affected area twice daily on the weekdays only to prevent tachyphylaxis. He was also prescribed fluconazole 150 mg orally once weekly for four weeks. On follow-up, the lesions had reduced in size without resolution. Triamcinolone was switched to clobetasol 0.05% topical ointment to apply to the affected areas twice daily Monday through Friday. 

Outcome and follow-up

Long-term treatment options were discussed with the patient, who elected to continue topical steroid treatment only during his three-month follow-up visit. He stated he was compliant with treatment but only noticed a mild improvement in the lesion size. Other treatment options were discussed to include anti-neutrophil therapy, i.e., dapsone or colchicine. Both treatments have been shown to improve lesions in 3/15 patients reported to have NFE [6]. The patient declined to modify his treatment regime. He will continue to be followed monthly in the dermatology clinic.

## Discussion

NFE is a rarely described neutrophil-predominant figurate erythema, with only 15 cases described to date. NFE can occur in both adults and children, known in pediatric populations as NFE of infancy [1-3]. Given its rarity, it has not yet been classified as a variant EAC or as a distinct neutrophilic dermatosis. As NFE may be a clinical variant of EAC, it may be valuable to determine a specific trigger of disease, which if avoided may induce disease resolution or prevent recurrence. Identified causes of EAC include hypersensitivity reaction, or it may be associated with other systemic infections (i.e., tinea, HIV, syphilis), drugs, neoplasms, etc. This patient did not have any evidence of systemic infection or malignancy on laboratory testing or by review of systems. He also denied taking any medications regularly. The patient was deployed on a ship at the time of disease presentation, which may suggest a possible hypersensitivity reaction to an unknown antigen. However, the lesions did not improve when he was removed from the ship to a familiar environment. This patient did not experience improvement in his lesion size with topical steroids and may have benefited from a trial of anti-neutrophil therapy.

## Conclusions

NFE is an annular erythema that has been rarely described in adults and typically has a highly variable duration, with most cases ranging from several days to decades. Despite the clinical similarity to other figurate erythemas, histopathology often reveals perivascular neutrophils and eosinophils in the dermis. NFE can mimic common fungal infections, and fungal etiologies should be ruled out prior to diagnosis. NFE is a poorly understood entity. It is important to keep NFE on the differential for non-resolving annular rashes, as patients may benefit from anti-neutrophil therapy.

## References

[1] Ahn CS, Huang WW (2022). Erythema annulare centrifugum and other figurate erythemas. Fitzpatrick's Dermatology.

[2] Hamidi S, Prose NS, Selim MA (2019). Neutrophilic figurate erythema of infancy: a diagnostic challenge. J Cutan Pathol.

[3] Patrizi A, Savoia F, Varotti E, Gaspari V, Passarini B, Neri I (2008). Neutrophilic figurate erythema of infancy. Pediatr Dermatol.

[4] M. Tye Haeberle, MD MD (2022). Erythema annulare centrifugum. https://www.uptodate.com/contents/erythema-annulare-centrifugum.

[5] Bolognia JL, Jorizzo JL, Rapini RP (2012). Figurate erythemas. Dermatology.

[6] Wu YH, Hsiao PF (2017). Neutrophilic figurate erythema. Am J Dermatopathol.

[7] Ozdemir M, Engin B, Toy H, Demirkesen C (2008). Neutrophilic figurate erythema. Int J Dermatol.

[8] Ghosh SK, Bandyopadhyay D, Haldar S (2012). Neutrophilic figurate erythema recurring on the same site in a middle-aged healthy woman. Indian J Dermatol Venereol Leprol.

[9] Kim DH, Lee JH, Lee JY, Park YM (2016). Erythema annulare centrifugum: analysis of associated diseases and clinical outcomes according to histopathologic classification. Ann Dermatol.

[10] Adler DJ, Rower JM, Hashimoto K (1981). Annular pustular psoriasis. Arch Dermatol.

